# The Impact of Dietary Glycemic Index and Glycemic Load on Postprandial Lipid Kinetics, Dyslipidemia and Cardiovascular Risk

**DOI:** 10.3390/nu12082204

**Published:** 2020-07-24

**Authors:** Vaia Lambadiari, Emmanouil Korakas, Vasilios Tsimihodimos

**Affiliations:** 1 Second Department of Internal Medicine and Research Institute, University General Hospital Attikon, 124 62 Haidari, Greece; mankor-th@hotmail.com; 2 Department of Internal Medicine, School of Medicine, University of Ioannina, 451 10 Ioannina, Greece; vtsimi@uoi.gr

**Keywords:** glycemic index, glycemic load, carbohydrates, postprandial hypertriglyceridemia, cardiovascular disease, lipids, fructose

## Abstract

Many recent studies have acknowledged postprandial hypetriglyceridemia as a distinct risk factor for cardiovascular disease. This dysmetabolic state is the result of the hepatic overproduction of very low-density lipoproteins (VLDLs) and intestinal secretion of chylomicrons (CMs), which leads to highly atherogenic particles and endothelial inflammation. Postprandial lipid metabolism does not only depend on consumed fat but also on the other classes of nutrients that a meal contains. Various mechanisms through which carbohydrates exacerbate lipidemia have been identified, especially for fructose, which stimulates de novo lipogenesis. Glycemic index and glycemic load, despite their intrinsic limitations, have been used as markers of the postprandial glucose and insulin response, and their association with metabolic health and cardiovascular events has been extensively studied with contradictory results. This review aims to discuss the importance and pathogenesis of postprandial hypertriglyceridemia and its association with cardiovascular disease. Then, we describe the mechanisms through which carbohydrates influence lipidemia and, through a brief presentation of the available clinical studies on glycemic index/glycemic load, we discuss the association of these indices with atherogenic dyslipidemia and address possible concerns and implications for everyday practice.

## 1. Introduction

Cardiovascular disease (CVD) is the leading cause of death in the Western world [1]. Metabolic derangement has long been identified as a predisposing factor for CVD outcomes, and a major component of it is dyslipidemia. For years, fasting values of blood lipids have been in the spotlight of research [2]. However, in the Western countries, most individuals consume fat-containing meals, snacks and drinks at regular 4 to 5 h intervals, and, therefore, they are considered to be in a, nearly constant, postprandial state. Postprandial hypertriglyceridemia contributes to atherogenic dyslipidemia (AD), a term used to describe the combination of plasma hypertriglyceridemia, low high-density lipoprotein cholesterol (HDL-C) and increased small and dense low-density lipoprotein cholesterol (LDL-C) particles [3]. This dysmetabolic state is the result of (a) the excessive postprandial accumulation of triglyceride-rich lipoprotein (TRL) particles through two distinct secretory pathways—the production of very-low-density lipoproteins (VLDLs) in the liver and secretion of chylomicrons (CMs) in the small intestine—and (b) defective TRL clearance from the circulation [4]. Regardless of the mechanism, postprandial hypertriglyceridemia is now considered a distinct risk factor for CVD, and it has been associated with a number of factors such as dietary patterns, physical activity, gene polymorphisms, insulin resistance, obesity and other dysmetabolic conditions [5].

Postprandial lipemia and, in general, postprandial metabolic responses are highly dependent both on the quality and the quantity of the food consumed. The glycemic index (GI) was introduced in 1981 by Jenkins et al. as a ranking system for carbohydrates based on their potential to elevate glucose levels postprandially, and it was originally designed as a tool for people with diabetes mellitus (DM) to select foods that would cause the least possible exacerbation of postprandial glycemia [6]. However, it soon became clear that the glucose response could not be attributed only to GI; if large amounts of food with a low GI were consumed, the glucose response would still be high. To address this discrepancy, the concept of the glycemic load (GL) was introduced in 1997 as a means of predicting the glucose response to a specific food, calculated by multiplying the food’s GI by the grams of carbohydrate in the food [7]. Despite many confounding factors in the estimation of the GI and GL, many studies in recent years have pointed out their association with metabolic diseases and CVD [8]. In this review, we will describe the association of postprandial hypertriglyceridemia with CVD, and we will discuss the available clinical studies on GI, GL and cardiovascular risk factors. Then, we will discuss the pathophysiological mechanisms through which postprandial lipemia is affected by dietary components and how it is associated with GI/GL through the available studies on the field.

## 2. Postprandial Hypertriglyceridemia as a Risk Factor for Cardiovascular Disease

Hypertriglyceridemia has for many years been questioned as a CVD risk factor. This is partly due to the high within-person variability compared to that of other lipoproteins, which made it a factor difficult for which to prove causality in clinical trials. However, in the recent years, the residual CVD risk that remains after the optimization of statin therapy has been largely attributed to so-called atherogenic dyslipidemia, which is the combination of hypertriglyceridemia and low HDL-C often seen in patients with metabolic syndrome and insulin resistance [9]. We usually assess the correlation of CVD risk and hypertriglyceridemia using the fasting values. However, in Western societies, we spend most of our daytime in the postprandial state. Mechanistic studies with mixed meals have shown that after meal ingestion, triglycerides continue to rise for 5 to 6 h and that the slope of the curve depends on the baseline values and the rate of insulin sensitivity [10,11]. In the presence of insulin resistance, metabolic syndrome and type 2 diabetes, triglycerides are high both in the fasting and in the postprandial state. The same stands for other atherogenic triglyceride-rich lipoproteins, such as VLDL and chylomicrons. Triglycerides serve as an index for all these lipoproteins that contribute to CVD risk and reflect postprandial lipid kinetics [9,12,13]. All ApoB-containing lipoproteins <70 nm in diameter, i.e., small TG-rich lipoproteins and their remnants, can cross the endothelial barrier, where they can become trapped after interaction with extracellular structures such as proteoglycans. Endothelial dysfunction and increased vascular permeability exacerbate the latter [14]. This process leads to lipid accumulation on the arterial wall and atherogenesis. High plasma circulating Apo-B-containing lipoproteins, as reflected to a large extent by the fasting and non-fasting plasma triglyceride values, induce more severe atherosclerosis, with larger plaques, especially if these high concentrations stand for long periods of time. They also seem to act synergistically with LDL particles [2,15].

The risk of an acute atherosclerotic CVD event is strongly correlated to the plasma level of ApoB-containing lipoproteins. Thus, it would be reasonable to assume that lowering these levels could have a preventive impact on CVD [2]. Large randomized controlled trials (RCTs) have clarified the contribution of triglycerides to the residual CVD risk. In particular, the ACCORD-Lipid trial studied a specific population of 5518 patients with diabetes and CVD in terms of the optimal treatment of the other risk factors. After adding fenofibrate to simvastatin treatment, there was a reduction on microalbuminuria and macroalbuminuria but not in the primary outcome of fatal cardiovascular events, non-fatal myocardial infarction, or non-fatal stroke compared to with simvastatin monotherapy. However, in the subgroup of patients with mixed atherogenic dyslipidemia (which accounted for 17% of the study population), fenofibrate reduced the CVD risk by 31% [16]. Comparable were the results from the Fenofibrate Intervention in Event Lowering in Diabetes (FIELD) trial. Fenofibrate treatment reduced total cardiovascular events by 27% in patients with diabetic dyslipidemia (triglyceride > 2.3 mmol/L, HDL-C> <1 mmol/L) and by 23% in patients with hypertriglyceridemia alone, an effect that was depicted in a systematic review by Maki et al. [17], where subgroup analyses from randomized controlled trials suggested a reduction in cardiovascular events in patients with elevated TGs, particularly if accompanied by low HDL-C. Diabetic microangiopathy was also improved [18]. A large series of studies with real-world evidence suggests that in patients treated optimally with statins but at high CVD risk, high triglycerides confer them a worse CVD and health economic status [19]. However, in spite of these results, the combination of statin and fibrate is not recommended as a standard therapy for mixed dyslipidemia. As the REDUCE-IT trial demonstrated, high dose omega-3 fatty acids in the form of icosapent ethyl at 4 g/day led to a 25% relative risk reduction for the primary end point composite of cardiovascular death, nonfatal myocardial infarction, nonfatal stroke, coronary revascularization or unstable angina, and a reduction in cardiovascular death of 20%. However, as similar results for the other types of omega-3 fatty acids are lacking, the clinical implications of this study are not to be extrapolated [20]. Indeed, when addressing triglycerides per se, the association of their level with atherosclerotic events disappears after adjustment for non-HDL-C, which reflects all circulating ApoB-containing lipoproteins. That is the rationale behind the use of non-HDL-C in patients with mixed atherogenic dyslipidemia rather than LDL-C or triglycerides solely to assess lipid targets [21]. Mendelian randomization studies also suggest that circulating ApoB-containing lipoproteins are correlated to atherosclerosis risk rather than triglycerides per se. However, measuring Apo-B particles is not easy in everyday practice, so triglycerides and non-HDL-C could act as good markers of atherogenic lipoprotein levels indirectly [22]. On the other hand, in atherogenic dyslipidemia, high triglycerides are accompanied by low HDL-C. However, to date, pharmacological approaches that increase HDL-C have not been proven to confer any benefit in terms of actual CVD outcomes. The latter was true for cholesteryl ester transfer protein (CETP) inhibitors such as dalcetrapid, anacetrapib and evacetrapid, or for nicotinic acid [23,24,25,26,27]. Thus, low HDL-C could also act as a marker rather than a pharmacological therapeutic target in patients with dyslipidemia. What is common, however, in most guidelines for diabetes, lipids or cardiovascular prevention is the importance of dietary intervention, with a reduction in saturated fats and/or simple carbohydrates to address dyslipidemia [21,28,29].

**Figure 1:**
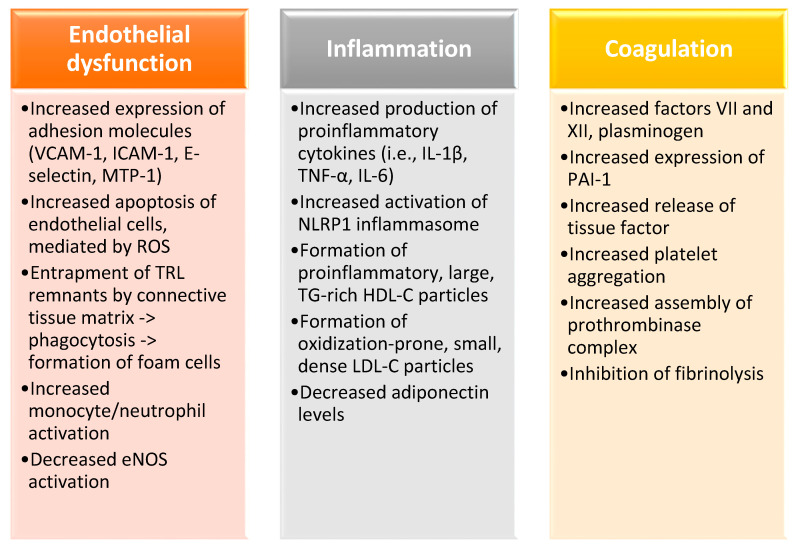
The main mechanisms through which hypertriglyceridemia leads to cardiovascular derangement are endothelial dysfunction, oxidative stress, chronic inflammation, and the induction of a hypercoagulative state. VCAM-1, vascular cell adhesion molecule 1; ICAM-1, intercellular adhesion molecule 1; MTP-1, microsomal triglyceride transfer protein 1; ROS, reactive oxygen species; TRL, triglyceride-rich lipoprotein; eNOS, endothelial nitric oxide synthase; IL-1β, interleukin 1β; TNF-α, tumor necrosis factor α; IL-6, interleukin 6; TG, triglycerides; HDL-C, high-density lipoprotein cholesterol; LDL-C, low-density lipoprotein cholesterol; PAI-1, plasminogen activation inhibitor-1.

Postprandial lipoprotein metabolism is a dynamic metabolic state, with a considerable within-day and inter-individual variability, and has a very complicated physiology. Postprandial VLDL and chylomicrons remnants affect endothelial function either through direct toxic effects or changes in the vascular tone [30]. In vivo studies assessing postprandial metabolism after high-fat meals have shown that post-challenge triglyceridemia negatively correlates with flow-mediated vasodilatation in healthy individuals [31]. Physiological studies with mixed meals showed that fasting and postprandial triglycerides were also negatively associated with the blood flow response in the subcutaneous adipose tissue of individuals across the whole spectrum of metabolic derangement, i.e., normoglycemic but insulin-resistant first-degree relatives of patients with diabetes, prediabetic patients and patients with diabetes but solely postprandial hyperglycemia, and, finally, patients with overt diabetes but treatment-naïve [32]. The same was true for blood flow responsiveness in the forearm skeletal muscles after a mixed meal in individuals within a broad range of metabolic derangement, where it mainly correlated with postprandial triglycerides and free fatty acids [11]. Postprandial lipemia has been associated with changes in hemostatic variables known to increase the risk of thrombotic events. Following the intake of a fat-rich meal, factor VIIc is transiently increased due to an increase in the plasma concentration of factor VIIa, which is a result of the increased TG and chylomicron levels [33]. A postprandial decline in plasminogen activator inhibitor type-1 activity and an increase in tissue plasminogen activator activity have been observed in various studies [34]. Finally, postprandial lipemia is associated with a mild increase in platelet reactivity that increases the expression of cell-surface markers in healthy men [35]. The main mechanisms through which hypertriglyceridemia has been shown to lead to cardiovascular derangement are summarized in Figure 1.

## 3. GI/GL: Definition, Measurement and Clinical Significance

### 3.1. Definition, Measurement and Limitations

The GI is defined as the incremental area-under-the-blood-glucose-curve (iAUC) after the consumption of a test food as a percentage of the AUC following the consumption of pure glucose (GI:100) by the same person on a different day. GL, as mentioned above, is calculated by multiplying the GI of a food by the total amount of carbohydrates in it [36]. According to the classification system applied internationally, foods are classified by GI into low (GI ≤ 55), medium (GI 56–69) and high (GI ≥ 70) categories, and by GL as being low (GL ≤ 10), medium (GL 11–19) and high (GL ≥ 20). Surprisingly, though, it has been noted that the GL is not directly proportional to the amount of food eaten. In a study by Brand-Miller et al., it was shown that the six-fold consumption of bread led to a three-fold increase in the glucose AUC [37]. In addition, the relationship between the GI and GL is not straightforward, as the glucose response postprandially can be attenuated when a high-GI food is eaten in small quantities, and vice versa. Apart from the type and quantity of the carbohydrates contained, the GI of foods depends on various other factors. Food processing such as gelatinization or retrogradation, which involve modifications in the structure of starches, or even cooking time and methods have all been shown to alter the postprandial glycemic response [38]. In a study by Brand et al. [39], where conventionally cooked, unprocessed foods and factory-processed foods were incubated for 3 h with human saliva and porcine pancreatin, a higher proportion of starch was digested in the factory-processed group, and a higher GI was produced when these foods were consumed by healthy individuals. In addition, the presence of fat or dietary fiber in the meal has been shown, although not consistently, to alter the postprandial glycemic response [40]. Above all, it has been argued that differences in the glycemic indices of individual foods are blurred when these foods are incorporated in a mixed meal, and there is an ongoing debate as to whether the sum of the individual GIs of the foods in a meal reliably reflects the total GI of the meal. This has been the case in the studies by Flint et al. [41] and Wolever et al. [42], where the real total GI of the meals studied was completely or partially different from the sum of each individual GI, respectively. Therefore, it is evident that the combination of different foods renders the accurate calculation of the GI of a mixed meal quite difficult and that, despite their proven worth in estimating the dietary value of foods, the GI and GL are not the sole factors that determine a healthy dietary regimen.

### 3.2. GI/GL, Metabolic Health and Cardiovascular Risk

In recent decades, a number of studies, both observational and interventional in nature, have been conducted regarding the association of GI and GL with different aspects of metabolic diseases and cardiovascular risk. Although some results have been favorable, especially in patients with insulin resistance (IR) and diabetes [43,44,45,46], other studies have failed to show important benefits [47,48,49,50,51]. A reason for this inconsistency, apart from the limitations of the GI and GL as they were described, has probably been the relatively small samples or the limited durations of some studies, which were inadequate for substantial metabolic changes to emerge. Below, a summary of the current data on the association of GI/GL with satiety, body weight, glucose homeostasis, lipids and cardiovascular risk is presented.

#### 3.2.1. GI/GL and Satiety

As the regulation of satiety is based on complex physiological pathways that entail hormones such as insulin, ghrelin, leptin, peptides such as glucagon-like peptide 1 (GLP-1) and peptide tyrosine-tyrosine (PYY) [52], the evaluation of the effects of the GI/GL has been mostly relied on subjective, self-reported measures. As for the acute effects of GI on satiety immediately in the postprandial period and up to 12 h post-meal, no significant associations have been identified [53]. Regarding the long-term effects of diets with different GI/GL on satiety (studies with a follow-up period from 4.5 days to 12 months), results have been contradictory. In two studies on overweight and obese adults, which lasted 3 and 4 weeks, respectively, the groups on the low-GI diet reported greater satiation and fewer food cravings [54,55]. On the contrary, in a randomized trial where 34 overweight adults followed controlled diets with low or high GL for 12 months, no differences in self-reported hunger or satiety were reported [56], and similar were the results in a 6-month weight loss intervention, where 122 overweight or obese adults were randomized to diets with different GI and fat content [57].

#### 3.2.2. GI/GL and Body Weight

Results from cross-sectional studies have not shown a significant effect of low GI or GL on body weight in terms of body mass index (BMI) or waist circumference, and among those that showed some benefit, the differences between extreme percentiles of BMI did not exceed 1.0 kg/m^2^ [53]. Regarding interventional studies, in a short-term study where 32 obese adults were randomly assigned to follow one of two either low- or high-GI, energy-restricted diets, the participants in the low-GI group showed greater reductions in body weight and BMI, an effect that was largely attributed to the greater fiber content of the low-GI diet [58]. Similarly, in a 6-month trial where 122 overweight and obese adults were randomized to one of three energy-restricted diets (-500 kcal/day) for 6 months, the BMI reduction was greater in the low GI group [57]. Contrary to these results, a 12-month intervention with 46 overweight adults on low and high GL diets with some degree of energy restriction reported no statistically significant differences in body weight parameters [59], and similar were the results of other long-term, highly controlled feeding interventions [60,61]. In a recent meta-analysis of 101 studies, low GI diets resulted in small but significant improvements in body weight; however, no individual control diet was significantly different from low GI diets [62].

#### 3.2.3. GI/GL and Glucose Homeostasis

Results from cross-sectional studies have failed to show a consistent association of GI/GL with glycemic status (Table 1). In a study by Wang et al. in 238 obese low-income Latino individuals [63], a positive association between GI and HbA1c was shown, while GL (but not GI) was associated with HbA1c and fasting glucose in a study by Farvid et al. in 640 adults with type 2 diabetes mellitus (T2DM) [64]. On the contrary, in a study by Mayer-Davis et al. in 1255 adults with or without IR or T2DM, no association of GI or GL with any index of glucose homeostasis was shown [65]. Earlier large-scale, prospective studies such as the Nurses’ Health Study and the Health Professionals Follow-Up Study demonstrated a positive association of GI with T2DM risk and supported the notion of the consumption of low-GI foods by diabetic patients as a healthy dietary regimen [66,67]; however, more recent studies such as the Atherosclerosis Risk in Communities Study (ARIC) [43] and research by Simila et al., Sluijs et al., Van Woudenbergh et al. and Sahyoun et al. [47,48,49,50], all with a substantial sample size, failed to show any relationship of GI or GL with any aspect of glucose control. Many well-controlled nutritional interventions in overweight or obese adults, despite producing favorable results in some aspects of glucose homeostasis, failed to demonstrate any serious differences between the different dietary regimens [53], except for the study by Runchey et al., where the low GL diet resulted in lower fasting glucose and insulin-like growth factor 1 (IGF-1) concentrations, a benefit which, however, applied only to those subjects with excessive body fat [68]. A recent meta-analysis by Ojo et al. indicated a significant improvement in fasting glucose and HbA1c with a low-GI diet; however, these results were mainly driven by the two out of the five studies analyzed [69]. In a meta-analysis of three large-scale RCTs by Bhupathiraju et al. [70], subjects in the highest quintile of energy-adjusted GL had a 10% higher risk of T2DM. In the hallmark meta-analysis by Barclay et al. [44], including 37 prospective cohort studies, a positive correlation with T2DM was demonstrated of high GI (RR: 1.40; 95% CI: 1.23–1.59) and high GL (RR: 1.27; 95% CI: 1.12–1.45), and similar results were reproduced in the meta-analysis by Dong et al. [71]. In a systematic review by Livesey et al. [72], including 24 prospective cohort studies, the GL was positively associated with an RR of T2D of 1.45 (95% CI: 1.31–1.61) for a 100 g increment in GL. In summary, despite a strong favorable trend, no robust association between indices of glycemic control and GI or GL has been demonstrated, and it seems that factors such as the different dietary components such as fiber or the degree of energy restriction in the diet are crucial in determining glycemic control.

#### 3.2.4. GI/GL and Cardiovascular Events

The majority of studies have focused on coronary heart disease (CHD); cardiovascular events, both fatal and non-fatal ones; and stroke (Table 2). In a study by Yu et al. in 117,366 Chinese women and men (40–74 years of age) without a history of diabetes, CHD, stroke, or cancer, a positive association of GL with the prevalence of CHD was shown [73]. On the contrary, two other large-scale studies showed no association of GI or GL with adverse cardiovascular outcomes [74,75]. In a study by Levitan et al. [76], where 36,246 Swedish men were recruited for a follow-up period of 6 years, no association of GI or GL with myocardial infarction, stroke or total CVD mortality was indicated, and similar were the results obtained by Burger et al. in 6192 adults with T2DM followed up for 9.2 years [77]. Other large-scale studies have also failed to show an association of GI or GL with stroke [54]. In a prospective, observational study of 36,019 women, 48–83 years old, without baseline heart failure (HF), diabetes or myocardial infarction, no association between GI and heart failure events was reported after 9 years of follow-up [78]. A meta-analysis by Shahdadian et al. [51] showed no significant association between dietary GI or GL and CVD mortality. On the other hand, meta-analyses by Barclay et al. [44] and Fan et al. [45] demonstrated a significant positive association between high GI and GL and coronary heart disease. In the same notion, in a meta-analysis including fourteen studies and 229,213 participants, the pooled RRs of CVD risk for the highest vs. lowest categories of GL and GI were 1.23 and 1.13, respectively [46]. Overall, data regarding the association between GI/GL and cardiovascular events are considered equivocal.

#### 3.2.5. GI/GL and Blood Lipids

The effects of GI/GL on fasting blood lipids are controversial. Levitan et al. measured the dietary glycemic indices (GIs) and dietary glycemic loads (GLs) among 18,137 healthy women with DM using a food-frequency questionnaire. Dietary GI was significantly associated with HDL-C, LDL-C, the LDL-C/HDL-C ratio and TG, and the same results applied to GL except for LDL-C [79]. Liese et al. studied 1026 middle-aged adults with normal or impaired glucose tolerance and showed a positive association of GL and carbohydrate consumption with total and LDL cholesterol and an inverse association with HDL-C, while, in women, associations were limited to triglycerides [80]. However, many other studies have failed to show a significant association of TC, LDL-C and HDL-C with GI or GL [81,82,83]. Regarding TGs, Shikany et al. showed a positive association of GL with triacylglycerols (TAGs) [84]. In a study in 1354 Japanese female farmers, Murakami et al. indicated a positive association of GI and GL with fasting TAGs [83]. In the studies of Hosseinpour-Niazi et al. [85] and McKeown et al. [86], dietary GI was positively associated with fasting TGs, with the differences between extreme percentiles ranging from 11 to 21 mg/dL.

## 4. Postprandial Lipemia and Carbohydrates: Pathophysiology and Available Clinical Data

### 4.1. Regulation of Postprandial Lipemia and Association with Dietary Carbohydrates

After a meal, dietary free fatty acids (FFAs) are absorbed from the gut and converted to triacylglycerols to be incorporated into triglyceride-rich apolipoprotein B-48 (apoB-48) chylomicrons (CMs) in the intestinal epithelial cells, which are then transferred into the blood via the lymphatic system. VLDL particles are triacylglycerol-rich apo B-100-containing particles, synthesized by the liver. Both VLDL and chylomicrons are hydrolyzed by lipoprotein lipase, and their remnants are removed from the circulation via hepatic receptors [4]. Chylomicrons, VLDL and their respective remnants (remnant lipoproteins) are termed triacylglycerol-rich lipoproteins (TRLs) [30]. The postprandial hyperinsulinemia normally suppresses lipolysis and hepatic VLDL production. However, in cases of impaired insulin sensitivity, these actions are blunted, an effect that eventually leads to the creation of smaller, dense LDL-C particles with a high atherogenic potential and smaller, TRL-rich HDL particles that are rapidly removed from the circulation. The rate at which lipids are absorbed and cleared depends on various factors such as metabolic diseases and the genetic background of an individual, along with lifestyle choices such as the rate of physical activity and the composition and structure of the foods consumed [30]. The main factor seems to be the amount of total fat, as high-fat meals have exhibited a greater degree of postprandial lipemia in some studies [40]. The effects of different fatty acids (FAs) on postprandial hypetriglyceridemia are rather contradictory. Studies have either shown no differences among fatty acids or lower plasma triglyceride iAUC after the consumption of either saturated fatty acid (SFA)-rich meals or n-6 polyunsaturated fatty acid (n-6 PUFA)-rich meals [87,88,89,90]. Apart from fat, however, the other classes of macronutrients—namely, carbohydrates and protein—also exert an impact on lipid metabolism [40].

The addition of glucose or digestible oligosaccharides to a fatty meal results in a delay in gastric emptying [91,92]. More importantly, regarding lipid digestion, high levels of digestible carbohydrates in the diet attenuate the lipase secretion by the gastric mucosa or the pancreas into the small intestine [93,94], while indigestible carbohydrates, i.e., dietary fibers, can lower the extent of lipolysis either through the reduction of lipid emulsification or through forming aggregates with lipid globules [95,96]. In addition, carbohydrates can intervene in intestinal lipid absorption and intracellular lipid processing. A high extracellular glucose concentration increased brush border membrane fluidity and permeability in human intestinal Caco-2 cells and isolated loops of the small intestine, thus affecting intestinal lipid uptake [97,98]. High glucose levels downregulate a protein kinase C (PKC) pathway that regulates cholesterol absorption and uptake from HDL-C and decreases lipid accumulation in human macrophages, while they increase the mRNA levels of lipid transporters such as ATP-binding cassette transporter ABCA1 and the microsomal triglyceride transfer protein (MTP), which results in higher levels of intestinal triglyceride-rich apoB-48-containing lipoproteins [99,100,101].

A special mention needs to be made of a distinct mechanism exerted by fructose. Normally, hepatic lipogenesis accounts for only a minimal degree of the de novo TG synthesis in humans. However, unlike glucose, fructose is preferentially metabolized by the liver and stimulates de novo lipogenesis (DNL) [79]. Upon entering the liver, fructose is phosphorylated at the 1-position by ketohexokinase to form fructose-1-phosphate [86]. The fructose-1-phosphate is readily converted to triose phosphates, which provide the backbone for triglycerides. The hepatic metabolism of ingested fructose increases levels of intrahepatic carbohydrate metabolites, such as carbohydrate response element-binding protein (ChREBP), which promotes glycolysis and lipogenesis [102], and sterol regulatory element-binding protein 1c (SREBP1c), which facilitates the storage of fatty acids as triglycerides [103]. Fructose also increases acetyl-CoA concentrations in the liver, subsequently leading to the increased production of malonyl-CoA. This inhibits the entry of fatty acids into the mitochondria and contributes to lipogenesis through the binding of acetyl-CoA to the long-chain fatty acids, providing carbon atoms for both glycerol and the acyl portion of the acylglycerol molecule [104]. In general, fructose reduces adipose tissue lipolysis [105], increases FA clearance, increases VLDL remnant concentrations [106] and promotes a shift to energy sourcing by promoting carbohydrate oxidation through complex pathways [107], which ultimately leads to both fasting and postprandial hypertriglyceridemia and non-alcoholic fatty liver disease (NAFLD), effects that are amplified in a hyperinsulinemic, insulin-resistant environment.

Often considered the hepatic manifestation of the metabolic syndrome, NAFLD is characterized by excessive intrahepatic lipid accumulation and is associated with the overconsumption of dietary sugars, which promote hepatic de novo lipogenesis (DNL) [108]. As high fructose corn syrup (HFCS) is frequently used as a sweetener and the intake of sugar-sweetened beverages reflects total fructose intake, a possible association between NAFLD and fructose, a low-GI monosaccharide (GI:19), has been suggested [109]. Ma et al. showed a positive co-relation of fatty liver disease to sugar-sweetened beverage consumption, an effect that was dose-dependent [110]. Similarly, in a study comparing patients with biopsy-proven NAFLD and controls, the consumption of fructose in the patient group was nearly 2- to 3-fold higher than that in controls [111]. On the contrary, data from a large Finnish cohort did not support an association between fructose intake and NAFLD [112], while in the study by Abdelmalek et al., daily fructose consumption was associated with lower steatosis grade and higher fibrosis stage [113]. Two meta-analyses from four isocaloric fructose- vs. glucose-intervention trials suggested that fructose per se does not contribute to NAFLD during short-term (1–8 weeks) studies [114,115]; however, this association emerged when hypercaloric fructose was compared to a weight-maintenance control diet, and similar were the results in other studies [116], suggesting that a reduction in fructose intake could be a potential therapeutic target.

### 4.2. GI/GL and Postprandial Hypertriglyceridemia: A Well-Established Association(?)

Many studies have shown a significant co-relation of dietary carbohydrate intake to postprandial lipemia (Table 3). In a study by Matikainen et al. [117], the consumption of a fructose-sweetened (high GL) diet for 12 weeks led to a substantial increase in the postprandial TG response. Similarly, in the study by Swarbrick et al. [118], a high GL intervention diet, for 10 weeks, led to increased 14 h postprandial TAG profiles. Stanhope et al. [119] showed, in overweight and obese subjects, that the 8-week consumption of fructose-sweetened beverages significantly lowered glucose and insulin post meal excursions and increased postprandial TG compared with the baseline diet and with the consumption of glucose-sweetened beverages, an effect that was attributed not only to the high GL of the beverages but also to the suppression of lipoprotein lipase (LPL) activity by fructose. This mechanism explains the results of a randomized, crossover study, where 14 subjects were given a fructose or glucose test meal after an overnight fast; at 4 h postprandially, newly synthesized fatty acids from fructose made up approximately 0.4% of circulating VLDL-triacylglycerol and newly synthesized triacylglycerol-glycerol made up 38%, contrary to the almost null contribution of glucose [120]. A similar notion was observed in a study by Bantle et al. [121], where a diet providing 17% of energy as fructose for 6 weeks induced significantly higher fasting, postprandial, and daylong plasma triacylglycerol concentrations than did an isoenergetic glucose diet (although only in the male subjects), and in another study where overweight and obese subjects consumed glucose- or fructose-sweetened beverages providing 25% of energy requirements for 10 weeks [122]. In a study in nine obese subjects with insulin resistance but normal triacylglycerolemia, the consumption of a high GI mixed meal, compared with a low GI one, increased the postprandial rise in plasma insulin and the accumulation of TRL-apoB-48 and TRL-apoB-100 at 4 and 2 h postprandially, respectively, thus increasing postprandial TG concentrations [123]. Harbis et al. showed a strong positive correlation during the 6 h postprandial period between the apoB-48 plasma concentration and insulin plasma concentration, which was obviously higher with higher glycemic index meals [124]. In a second study, using a 3 h hyperinsulinemic–euglycemic clamp, an early reduction in apoB-48 concentration was followed by a late accumulation of plasma apoB-48 and triglycerides, indicating again the positive correlation of hyperinsulinemia with postprandial lipemia in non-insulin-resistant subjects. In the report by Bouché et al. [125], the plasma triacylglycerol excursion after lunch was substantially lowered in the low-glycemic index (LGI) group. As fasting triacylglycerolemia is a known determinant of postprandial triacylglycerolemia, the above-mentioned studies [79,80,83,84,85,86] where a low GI/GL showed beneficial effects on fasting triglyceride levels could also be considered to support the association of glycemic index and glycemic load with postprandial lipemia in an indirect way.

On the other hand, some studies have questioned this association and have raised some serious concerns (Table 4). In a study by Bukkapatnam et al. in 15 healthy postmenopausal women [126], triglyceride levels for the first 4 h after the meal were significantly lower in the high-glycemic index meal group, despite the concomitant higher insulin levels and Homeostatic Model Assessment for Insulin Resistance (HOMA-IR). In a randomized, controlled, single-blinded crossover study in 20 healthy Chinese men, the incremental area under the curve (iAUC) for TG was significantly lower after the SFA and PUFA meals than after the MUFA meal, irrespective of GI [127]. Despland et al. [128] found no difference in postprandial triglycerides in eight healthy males consuming a diet containing 25% of the energy as honey or pure fructose–glucose compared to an isocaloric starch diet. Similar results were indicated in a study by Campos et al., where the substitution of high sugar-sweetened beverages by artificially sweetened beverages for 12 weeks did not decrease postprandial TG despite the lower energy and fructose content of the meals [129]. Even more importantly, in a meta-analysis by Livesey et al. [130], a daily intake of fructose above 50 g and 100 g was required to exert any impact on postprandial and fasting TGs, respectively. It therefore becomes evident that the isocaloric inclusion of fructose in mixed meals has inconsistent effects on postprandial TGs despite being a potent stimulant of de novo lipogenesis and that maybe the induction of postprandial hypertriglyceridemia takes place only when fructose is administered in high doses that supplement the diet with excess energy, a theory that was supported by the meta-analysis by Wang et al. [131]. Another serious concern is that in many of the studies conducted, the administered dose of fructose greatly surpassed the average daily intake of 60–70g, a fact that brings into doubt the applicability of these results to everyday dietary regimens [104]. These limitations and discrepancies among the studies do not confute the relationship of the glycemic index and glycemic load with postprandial lipemia; however, they suggest that the lipidemic response is also dependent upon other dietary and metabolic factors.

## 5. Conclusions

As modern dietary habits have led individuals to spend most of their daytime in a postprandial state, the role of postprandial hypertriglyceridemia in the pathogenesis of cardiovascular disease has been highlighted in recent years. Postprandial lipemia is characterized by an increase in TRL-rich lipoprotein levels, and although these phenomena have been traditionally attributed to dietary fat, it is now evident that other dietary components, such as carbohydrates, have a role to play. The glycemic index and glycemic load of food, despite their intrinsic limitations and their dependence on a number of different dietary, genetic and metabolic factors, have been widely used as a guide for a healthier dietary pattern. The results regarding their association with aspects of metabolic disease and cardiovascular health, however, have been conflicting, and the possible reasons for these discrepancies include the observational nature and limited duration of most studies, the relatively small samples and, above all, the physiological complex interplay between different factors influencing nutrient digestion and metabolism. High GI and GL have been considered to exacerbate postprandial hypertriglyceridemia, especially when fructose was administered, but contradictions have also been observed when lower doses were used or due to different food components and metabolic conditions such as insulin resistance. In conclusion, postprandial hypertriglyceridemia seems to be a promising therapeutic target, and glycemic index and glycemic load can be utilized as a useful tool for designing a generally healthy dietary regimen, but they should not be the sole factor to be taken into account for nutritional recommendations. Large randomized clinical trials and well-controlled feeding interventions are necessary to elucidate the effect of these indices on the postprandial metabolic state and assess the role of all the possible confounding factors so as to generate robust clinical guidelines, feasible for everyday, real-world practice.

## Figures and Tables

**Table 1:** Major studies and meta-analyses on the association of glycemic index and glycemic load with glucose homeostasis. T2DM, type 2 diabetes mellitus; GI, glycemic index; GL, glycemic load; HbA1c, glycosylated hemoglobin A1c; FSG, fasting serum glucose; CHO, carbohydrates; CHD, coronary heart disease; DM, diabetes mellitus; RR, relative risk;IR, insulin resistance.

Study	Type of Study	Sample	Results
Wang et al. [63]	Cross-sectional	238 low income Latino adults w/T2DM, 45–67 years, 33–36 kg/m^2^	Positive association between GI and HbA1c (but not GL)
Farvid et al. [64]	Cross-sectional	640 adults w/T2DM, 28–75 years	Positive association between GL and FSG, HbA1c No association of GI and HbA1c or FSG
Mayer-Davis et al. [65]	Cross-sectional	1255 adults with/without IR or T2DM, 55.3 years, 29.1 kg/m^2^	No association of GI/GL with glucose homeostasis
Hodge et al. [66]	Prospective	36,787 men and women aged 40–69 years without diabetes	Low GI and high CHO intake—> decreased risk of DM
Salmeron et al. [67]	Prospective	42,759 men without DM or cardiovascular disease, 40–75 years, 6 years of follow-up	Positive association between high GI and incidence of DM (RR: 1.37; 95% CI, 1.02–1.83). Positive association of high GL/low cereal fiber intake and incidence of DM (RR: 2.17, 95% CI; 1.04–4.54)
Hardy et al. [43]	Sub-analysis	13,051 individuals aged 45–64 years from the Atherosclerosis Risk in Communities (ARIC) study	High GI—> increased risk of CHD in African Americans High GL—> increased risk of CHD in Whites (without DM)
Simila et al. [47]	Prospective	25,943 male smokers, 50–69 years	No association of GI/GL with glucose homeostasis
Sluijs et al. [48]	Prospective	37,843 Netherlands adults, 21–70 years	No association of GI/GL with glucose homeostasis
Van Woudenbergh et al. [49]	Prospective	4366 Netherlands adults, ≥55 years	No association of GI/GL with glucose homeostasis
Sahyoun et al. [50]	Prospective	1898 adults, 70–79 years	No association of GI/GL with glucose homeostasis
Bhupathiraju et al. [70]	Meta-analysis	74,248 women from the Nurses’ Health Study, 90,411 women from the Nurses’ Health Study II, and 40,498 men from the Health Professionals Follow-Up Study	Positive association of high GI (RR: 1.19; 95% CI: 1.14–1.24) and GL (RR: 1.13; 95% CI: 1.08–1.17) with T2DM
Barclay et al. [44]	Meta-analysis	37 prospective cohort studies	Positive association of high GI (RR: 1.40; 95% CI: 1.23–1.59) and GL (RR: 1.27; 95% CI: 1.12–1.45) with T2DM
Dong et al. [71]	Meta-analysis	13 prospective cohort studies	Positive association of high GI (RR: 1.16; 95% CI: 1.06–1.26) and GL (RR: 1.20; 95% CI: 1.11–1.30) with T2DM
Livesey et al. [72]	Meta-analysis	24 prospective cohort studies	Positive association of GL with T2D (RR: 1.45 for a 100 g increment in GL; 95% CI: 1.31–1.61)

**Table 2:** Major studies and meta-analyses on the association of glycemic index and glycemic load with cardiovascular disease. CHD, coronary heart disease; F/U, follow-up; GL, glycemic load; GI, glycemic index; T2DM, type 2 diabetes mellitus; HF, heart failure; CVD, cardiovascular disease; RR, relative risk.

Study	Type of Study	Sample	Results
Yu et al. [73]	Prospective	117,366 Chinese adults; 40–74 years; without history of diabetes, CHD, stroke or cancer; F/U of 9.8 years for women, 5.4 years for men	Positive association of GL and CHD
Burger et al. [74]	Prospective	8855 men, 10,753 women, 21–64 years, F/U of 11.9 years	No association between GI/GL and CVD
Sieri et al. [75]	Prospective	44,132 adults, F/U of 7.9 years	No association between GI/GL and CHD
Levitan et al. [76]	Prospective	36,246 Swedish men, 45–79 years, F/U of 6 years	No association between GI/GL and CVD mortality
Burger et al. [77]	Prospective	6192 adults with T2DM, F/U of 9.2 years	No association between GI/GL and CVD mortality
Levitan et al. [78]	Prospective	36,019 women, 48–83 years, F/U of 9 years	No association between GI/GL and HF
Shahdadian et al. [51]	Meta-analysis	18 cohort studies, 251,497 participants	No association between GI/GL and CVD mortality
Barclay et al. [44]	Meta-analysis	37 prospective cohort studies	Positive association of GI with CHD (RR: 1.25; 95% CI: 1.00–1.56)
Fan et al. [45]	Meta-analysis	15 prospective cohort studies, 438,073 participants	Positive association of GL with CHD (RR: 1.49; 95% CI: 1.27−1.73), only in women Positive association of GL with stroke (RR: 1.19; 95% CI: 1.00−1.43)
Ma et al. [46]	Meta-analysis	14 prospective cohort studies, 229,213 participants	Positive association of GI (RR: 1.13; 95% CI: 1.04–1.22) and GL (RR: 1.23; 95% CI: 1.11–1.36) with CVD, both associations stronger for women

**Table 3:** Major studies favoring the association of GI and GL with postprandial lipemia. GL, glycemic load; TAG, triacylglycerol; TG, triglycerides; iAUC, incremental area-under-the-curve; VLDL, very low-density lipoprotein; DNL, de novo lipogenesis; TRL, triglyceride lipoproteins; apoB-100, apolipoprotein B-100; apoB-48, apolipoprotein B-48.

Study	Type of Study	Sample	Results
Matikainen et al. [117]	Prospective	66 obese men consumed fructose-sweetened beverages containing 75 g fructose/day (high GL) for 12 weeks	Increased postprandial TGs
Swarbrick et al. [118]	Prospective	7 overweight or obese postmenopausal women on high GL intervention diet, which included a fructose-sweetened beverage with each meal, for 10 weeks	14 h postprandial TAG profiles were significantly increased (iAUC 141% higher)
Stanhope et al. [119]	Prospective	Overweight and obese subjects, 8-week consumption of fructose-sweetened beverages	Increased postprandial TGs
Chong et al. [120]	Crossover	14 subjects, fructose or glucose test meal after an overnight fast	At 4 h postprandially, newly synthesized fatty acids from fructose = 0.4% of circulating VLDL-triacylglycerol, newly synthesized triacylglycerol-glycerol = 38%, newly synthesized fatty acids and triacylglycerol-glycerol from glucose = none of VLDL-triacylglycerol
Bantle et al. [121]	Prospective	24 healthy adult volunteers, diet with 17% of energy as fructose or diet sweetened with glucose, for 6 weeks	Higher fasting, postprandial, and daylong plasma triacylglycerol concentrations with fructose
Stanhope et al. [122]	Prospective	Overweight and obese subjects consumed glucose- or fructose-sweetened beverages providing 25% of energy requirements for 10 weeks	Fructose increased postprandial TGs and DNL
Harbis et al. [123]	Crossover	9 obese subjects with insulin resistance randomly ingested 2 test meals with different quantities of slowly available glucose	High GI meal increased accumulation of TRL-apoB-48 and TRL-apoB-100 at 4 and 2 h postprandially, respectively
Harbis et al. [124]	Crossover	10 healthy men, 4 isolipidic meals with various GIs	Positive association of GI and apoB-48 plasma concentration at 6 h postprandially
Bouché et al. [125]	Prospective	11 healthy men, 5-week low GI diet versus high GI diet	Low GI diet lowered plasma triacylglycerol excursion after lunch

**Table 4:** Major studies and meta-analyses that suggest no association between glycemic index/glycemic load and postprandial lipemia. TG, triglycerides; GI, glycemic index; iAUC, incremental area-under-the-curve.

Study	Type of Study	Sample	Results
Bukkapatnam et al. [126]	Crossover	15 healthy postmenopausal women, low or high GI meal	Increased postprandial TGs with low GI meal
Sun et al. [127]	Crossover	20 healthy Chinese men, isocaloric meals different in carbohydrate and fat quality, in random order	No association of GI with postprandial iAUC for TGs
Despland et al. [128]	Prospective	8 healthy males, diet containing 25% energy as honey or pure fructose–glucose compared to an isocaloric starch diet, for 8 days	No difference in postprandial triglycerides regardless of GI
Campos et al. [129]	Prospective	26 obese or overweight subjects, substitution of high sugar-sweetened beverages by artificially sweetened beverages for 12 weeks	No difference in postprandial triglycerides
Livesey et al. [130]	Meta-analysis	42 reports	Significant effects on postprandial triacylglycerols with intakes of ≥50 g fructose/day Significant effects are seen on fasting triacylglycerol with intakes of ≥100 g fructose/day
Wang et al. [131]	Meta-analysis	14 clinical trials	Fructose in isocaloric exchange for other carbohydrate does not increase postprandial TGs. Fructose providing excess energy increases postprandial TGs.

## Abbreviations

ABCA1	ATP-binding cassette transporter
AD	atherogenic dyslipidemia
ApoB	apolipoprotein B
AUC	area-under-the-curve
BMI	body mass index
CETP	cholesteryl ester transfer protein
CHD	coronary heart disease
ChREBP	carbohydrate response element-binding protein
CMs	chylomicrons
CVD	cardiovascular disease
DM	diabetes mellitus
DNL	de novo lipogenesis
FFAs	free fatty acids
GI	glycemic index
GL	glycemic load
GLP-1	glucagon-like peptide 1
HDL-C	high-density lipoprotein cholesterol
HF	heart failure
HOMA-IR	Homeostatic Model Assessment for Insulin Resistance
IGF-1	insulin-like growth factor 1
IR	insulin resistance
LDL-C	low-density lipoprotein cholesterol
LPL	lipoprotein lipase
mRNA	messenger ribonucleic acid
MTP	microsomal triglyceride transfer protein
MUFAs	monounsaturated fatty acids
NAFLD	non-alcoholic fatty liver disease
PKC	protein kinase C
PUFAs	polyunsaturated fatty acids
PYY	peptide tyrosine-tyrosine
SFAs	saturated fatty acid
SREBP1c	sterol regulatory element-binding protein 1c
T2DM	type 2 diabetes mellitus
TAGs	triacylglycerols
TGs	triglycerides
TRL	triglyceride-rich lipoprotein
VLDLs	very low-density lipoproteins

## References

[1] Cardiovascular Diseases (CVDs). https://www.who.int/news-room/fact-sheets/detail/cardiovascular-diseases-(cvds).

[2] Ference B.A., Graham I., Tokgozoglu L., Catapano A.L. (2018). Impact of Lipids on Cardiovascular Health: JACC Health Promotion Series. J. Am. Coll. Cardiol..

[3] Ferrari R., Aguiar C., Alegria E., Bonadonna R.C., Cosentino F., Elisaf M., Farnier M., Ferrières J., Filardi P.P., Hancu N. (2016). Current practice in identifying and treating cardiovascular risk, with a focus on residual risk associated with atherogenic dyslipidaemia. Eur. Heart J. Suppl. J. Eur. Soc. Cardiol..

[4] Nakajima K., Nakano T., Tokita Y., Nagamine T., Inazu A., Kobayashi J., Mabuchi H., Stanhope K.L., Havel P.J., Okazaki M. (2011). Postprandial lipoprotein metabolism: VLDL vs chylomicrons. Clin. Chim. Acta.

[5] Pirillo A., Norata G.D., Catapano A.L. (2014). Postprandial lipemia as a cardiometabolic risk factor. Curr. Med. Res. Opin..

[6] Jenkins D.J., Wolever T.M., Taylor R.H., Barker H., Fielden H., Baldwin J.M., Bowling A.C., Newman H.C., Jenkins A.L., Goff D.V. (1981). Glycemic index of foods: A physiological basis for carbohydrate exchange. Am. J. Clin. Nutr..

[7] Schwingshackl L., Hoffmann G. (2013). Long-term effects of low glycemic index/load vs. high glycemic index/load diets on parameters of obesity and obesity-associated risks: A systematic review and meta-analysis. Nutrition, metabolism, and cardiovascular diseases. Nutr. Metab. Cardiovasc. Dis..

[8] Desmarchelier C., Borel P., Lairon D., Maraninchi M., Valéro R. (2019). Effect of Nutrient and Micronutrient Intake on Chylomicron Production and Postprandial Lipemia. Nutrients.

[9] Watts G.F., Karpe F. (2011). Republished review: Triglycerides and atherogenic dyslipidaemia: Extending treatment beyond statins in the high-risk cardiovascular patient. Postgrad. Med. J..

[10] Dimitriadis G., Boutati E., Lambadiari V., Mitrou P., Maratou E., Brunel P., Raptis S.A. (2004). Restoration of early insulin secretion after a meal in type 2 diabetes: Effects on lipid and glucose metabolism. Eur. J. Clin. Investig..

[11] Lambadiari V., Mitrou P., Maratou E., Raptis A., Raptis S.A., Dimitriadis G. (2012). Increases in muscle blood flow after a mixed meal are impaired at all stages of type 2 diabetes. Clin. Endocrinol..

[12] Nordestgaard B.G., Benn M., Schnohr P., Tybjaerg-Hansen A. (2007). Nonfasting triglycerides and risk of myocardial infarction, ischemic heart disease, and death in men and women. JAMA.

[13] Sarwar N., Sandhu M.S., Ricketts S.L., Butterworth A.S., Di Angelantonio E., Boekholdt S.M., Ouwehand W., Kastelein J.J., Triglyceride Coronary Disease Genetics Consortium, Emerging Risk Factors Collaboration (2010). Triglyceride-mediated pathways and coronary disease: Collaborative analysis of 101 studies. Lancet.

[14] Tabas I., Williams K.J., Borén J. (2007). Subendothelial lipoprotein retention as the initiating process in atherosclerosis: Update and therapeutic implications. Circulation.

[15] Borén J., Williams K.J. (2016). The central role of arterial retention of cholesterol-rich apolipoprotein-B-containing lipoproteins in the pathogenesis of atherosclerosis: A triumph of simplicity. Curr. Opin. Lipidol..

[16] Ginsberg H.N., Elam M.B., Lovato L.C., Crouse J.R., Leiter L.A., Linz P., Friedewald W.T., Buse J.B., Gerstein H.C., ACCORD Study Group (2010). Effects of combination lipid therapy in type 2 diabetes mellitus. N. Engl. J. Med..

[17] Maki K.C., Dicklin M.R. (2017). Do triglyceride-lowering drugs decrease risk of cardiovascular disease?. Curr. Opin. Lipidol..

[18] Scott R., O’Brien R., Fulcher G., Pardy C., D’Emden M., Tse D., Taskinen M.R., Ehnholm C., Keech A., The FIELD Study Investigators (2009). Effects of fenofibrate treatment on cardiovascular disease risk in 9795 individuals with type 2 diabetes and various components of the metabolic syndrome: The Fenofibrate Intervention and Event Lowering in Diabetes (FIELD) study. Diabetes Care.

[19] Toth P.P., Fazio S., Wong N.D., Hull M., Nichols G.A. (2020). Risk of cardiovascular events in patients with hypertriglyceridaemia: A review of real-world evidence. Diabetes Obes. Metab..

[20] Bhatt D.L., Steg P.G., Miller M., Brinton E.A., Jacobson T.A., Ketchum S.B., Doyle R.T., Juliano R.A., Jiao L., Granowitz C. (2019). Cardiovascular Risk Reduction with Icosapent Ethyl for Hypertriglyceridemia. N. Engl. J. Med..

[21] Di Angelantonio E., Gao P., Pennells L., Kaptoge S., Caslake M., Thompson A., Butterworth A.S., Sarwar N., Wormser D., Emerging Risk Factors Collaboration (2012). Lipid-related markers and cardiovascular disease prediction. JAMA.

[22] Ference B.A., Kastelein J.J.P., Ray K.K., Ginsberg H.N., Chapman M.J., Packard C.J., Laufs U., Oliver-Williams C., Wood A.M., Butterworth A.S. (2019). Association of Triglyceride-Lowering LPL Variants and LDL-C-Lowering LDLR Variants with Risk of Coronary Heart Disease. JAMA.

[23] Frikke-Schmidt R., Nordestgaard B.G., Stene M.C., Sethi A.A., Remaley A.T., Schnohr P., Grande P., Tybjærg-Hansen A. (2008). Association of loss-of-function mutations in the ABCA1 gene with high-density lipoprotein cholesterol levels and risk of ischemic heart disease. JAMA.

[24] Schwartz G.G., Olsson A.G., Abt M., Ballantyne C.M., Barter P.J., Brumm J., Chaitman B.R., Holme I.M., Kallend D., Leiter L.A. (2012). Effects of dalcetrapib in patients with a recent acute coronary syndrome. N. Engl. J. Med..

[25] Voight B.F., Peloso G.M., Orho-Melander M., Frikke-Schmidt R., Barbalic M., Jensen M.K., Hindy G., Hólm H., Ding E.L., Johnson T. (2012). Plasma HDL cholesterol and risk of myocardial infarction: A mendelian randomisation study. Lancet.

[26] Boden W.E., Probstfield J.L., Anderson T., Chaitman B.R., Desvignes-Nickens P., Koprowicz K., McBride R., Teo K., Weintraub W., AIM-HIGH Investigators (2012). Niacin in patients with low HDL cholesterol levels receiving intensive statin therapy. N. Engl. J. Med..

[27] Bowman L., Hopewell J.C., Chen F., Wallendszus K., Stevens W., Collins R., Wiviott S.D., Cannon C.P., Braunwald E., HPS3/TIMI55–REVEAL Collaborative Group (2017). Effects of Anacetrapib in Patients with Atherosclerotic Vascular Disease. N. Engl. J. Med..

[28] Santos F.L., Esteves S.S., Da Costa Pereira A., Yancy W.S., Nunes J.P. (2012). Systematic review and meta-analysis of clinical trials of the effects of low carbohydrate diets on cardiovascular risk factors. Obes. Rev..

[29] Mach F., Baigent C., Catapano A.L., Koskinas K.C., Casula M., Badimon L., Chapman M.J., De Backer G.G., Delgado V., Ference B.A. (2020). 2019 ESC/EAS Guidelines for the management of dyslipidaemias: Lipid modification to reduce cardiovascular risk. Eur. Heart J..

[30] Lopez-Miranda J., Williams C., Lairon D. (2007). Dietary, physiological, genetic and pathological influences on postprandial lipid metabolism. Br. J. Nutr..

[31] Vogel R.A., Corretti M.C., Plotnick G.D. (1997). Effect of a single high-fat meal on endothelial function in healthy subjects. Am. J. Cardiol..

[32] Dimitriadis G., Lambadiari V., Mitrou P., Maratou E., Boutati E., Panagiotakos D.B., Economopoulos T., Raptis S.A. (2007). Impaired postprandial blood flow in adipose tissue may be an early marker of insulin resistance in type 2 diabetes. Diabetes Care.

[33] Miller G.J. (1998). Postprandial lipaemia and haemostatic factors. Atherosclerosis.

[34] Sanders T.A., Oakley F.R., Cooper J.A., Miller G.J. (2001). Influence of a stearic acid-rich structured triacylglycerol on postprandial lipemia, factor VII concentrations, and fibrinolytic activity in healthy subjects. Am. J. Clin. Nutr..

[35] Nordoy A., Strom E., Gjesdal K. (1974). The effect of alimentary hyperlipaemia and primary hypertriglyceridaemia on platelets in man. Scand. J. Haematol..

[36] Venn B.J., Green T.J. (2007). Glycemic index and glycemic load: Measurement issues and their effect on diet-disease relationships. Eur. J. Clin. Nutr..

[37] Brand-Miller J.C., Thomas M., Swan V., Ahmad Z.I., Petocz P., Colagiuri S. (2003). Physiological validation of the concept of glycemic load in lean young adults. J. Nutr..

[38] Glade M.J., Smith K. (2015). A glance at… glycemic index. Nutrition.

[39] Brand J.C., Nicholson P.L., Thorburn A.W., Truswell A.S. (1985). Food processing and the glycemic index. Am. J. Clin. Nutr..

[40] Dias C.B., Moughan P.J., Wood L.G., Singh H., Garg M.L. (2017). Postprandial lipemia: Factoring in lipemic response for ranking foods for their healthiness. Lipids Health Dis..

[41] Flint A., Møller B.K., Raben A., Pedersen D., Tetens I., Holst J.J., Astrup A. (2004). The use of glycaemic index tables to predict glycaemic index of composite breakfast meals. Br. J. Nutr..

[42] Wolever T.M., Yang M., Zeng X.Y., Atkinson F., Brand-Miller J.C. (2006). Food glycemic index, as given in glycemic index tables, is a significant determinant of glycemic responses elicited by composite breakfast meals. Am. J. Clin. Nutr..

[43] Hardy D.S., Hoelscher D.M., Aragaki C., Stevens J., Steffen L.M., Pankow J.S., Boerwinkle E. (2010). Association of glycemic index and glycemic load with risk of incident coronary heart disease among Whites and African Americans with and without type 2 diabetes: The Atherosclerosis Risk in Communities study. Ann. Epidemiol..

[44] Barclay A.W., Petocz P., McMillan-Price J., Flood V.M., Prvan T., Mitchell P., Brand-Miller J.C. (2008). Glycemic index, glycemic load, and chronic disease risk—A meta-analysis of observational studies. Am. J. Clin. Nutr..

[45] Fan J., Song Y., Wang Y., Hui R., Zhang W. (2012). Dietary glycemic index, glycemic load, and risk of coronary heart disease, stroke, and stroke mortality: A systematic review with meta-analysis. PLoS ONE.

[46] Ma X.Y., Liu J.P., Song Z.Y. (2012). Glycemic load, glycemic index and risk of cardiovascular diseases: Meta-analyses of prospective studies. Atherosclerosis.

[47] Simila M.E., Kontto J.P., Valsta L.M., Mannisto S., Albanes D., Virtamo J. (2012). Carbohydrate substitution for fat or protein and risk of type 2 diabetes in male smokers. Eur. J. Clin. Nutr..

[48] Sluijs I., Beulens J.W., Van Der Schouw Y.T., Van Der A.D., Buckland G., Kuijsten A., Schulze M.B., Amiano P., Ardanaz E., Balkau B. (2013). Dietary glycemic index, glycemic load, and digestible carbohydrate intake are not associated with risk of type 2 diabetes in eight European countries. J. Nutr..

[49] VanWoudenbergh G.J., Kuijsten A., Sijbrands E.J., Hofman A., Witteman J.C., Feskens E.J. (2011). Glycemic index and glycemic load and their association with C-reactive protein and incident type 2 diabetes. J. Nutr. Metab..

[50] Sahyoun N.R., Anderson A.L., Tylavsky F.A., Lee J.S., Sellmeyer D.E., Harris T.B. (2008). Dietary glycemic index and glycemic load and the risk of type 2 diabetes in older adults. Am. J. Clin. Nutr..

[51] Shahdadian F., Saneei P., Milajerdi A., Esmaillzadeh A. (2019). Dietary glycemic index, glycemic load, and risk of mortality from all causes and cardiovascular diseases: A systematic review and dose-response meta-analysis of prospective cohort studies. Am. J. Clin. Nutr..

[52] Choudhury S.M., Tan T.M., Bloom S.R. (2016). Gastrointestinal hormones and their role in obesity. Curr. Opin. Endocrinol. Diabetes Obes..

[53] Vega-López S., Venn B.J., Slavin J.L. (2018). Relevance of the Glycemic Index and Glycemic Load for Body Weight, Diabetes, and Cardiovascular Disease. Nutrients.

[54] Pal S., Lim S., Egger G. (2008). The effect of a low glycaemic index breakfast on blood glucose, insulin, lipid profiles, blood pressure, body weight, body composition and satiety in obese and overweight individuals: A pilot study. J. Am. Coll. Nutr..

[55] Chang K.T., Lampe J.W., Schwarz Y., Breymeyer K.L., Noar K.A., Song X., Neuhouser M.L. (2012). Low glycemic load experimental diet more satiating than high glycemic load diet. Nutr. Cancer.

[56] Das S.K., Gilhooly C.H., Golden J.K., Pittas A.G., Fuss P.J., Cheatham R.A., Tyler S., Tsay M., McCrory M.A., Lichtenstein A.H. (2007). Long-term effects of 2 energy-restricted diets differing in glycemic load on dietary adherence, body composition, and metabolism in calerie: A 1-y randomized controlled trial. Am. J. Clin. Nutr..

[57] Juanola-Falgarona M., Salas-Salvado J., Ibarrola-Jurado N., Rabassa-Soler A., Diaz-Lopez A., Guasch-Ferre M., Hernandez-Alonso P., Balanza R., Bullo M. (2014). Effect of the glycemic index of the diet on weight loss, modulation of satiety, inflammation, and other metabolic risk factors: A randomized controlled trial. Am. J. Clin. Nutr..

[58] Abete I., Parra D., Martinez J.A. (2008). Energy-restricted diets based on a distinct food selection affecting the glycemic index induce different weight loss and oxidative response. Clin. Nutr..

[59] Karl J.P., Cheatham R.A., Das S.K., Hyatt R.R., Gilhooly C.H., Pittas A.G., Lieberman H.R., Lerner D., Roberts S.B., Saltzman E. (2014). Effect of glycemic load on eating behavior self-efficacy during weight loss. Appetite.

[60] Buscemi S., Cosentino L., Rosafio G., Morgana M., Mattina A., Sprini D., Verga S., Rini G.B. (2013). Effects of hypocaloric diets with different glycemic indexes on endothelial function and glycemic variability in overweight and in obese adult patients at increased cardiovascular risk. Clin. Nutr..

[61] Sichieri R., Moura A.S., Genelhu V., Hu F., Willett W.C. (2007). An 18-mo randomized trial of a low-glycemic-index diet and weight change in Brazilian women. Am. J. Clin. Nutr..

[62] Zafar M.I., Mills K.E., Zheng J., Peng M.M., Ye X., Chen L.L. (2019). Low glycaemic index diets as an intervention for obesity: A systematic review and meta-analysis. Obes. Rev..

[63] Wang M.L., Gellar L., Nathanson B.H., Pbert L., Ma Y., Ockene I., Rosal M.C. (2015). Decrease in glycemic index associated with improved glycemic control among latinos with type 2 diabetes. J. Acad. Nutr. Diet..

[64] Farvid M.S., Homayouni F., Shokoohi M., Fallah A., Farvid M.S. (2014). Glycemic index, glycemic load and their association with glycemic control among patients with type 2 diabetes. Eur. J. Clin. Nutr..

[65] Mayer-Davis E.J., Dhawan A., Liese A.D., Teff K., Schulz M. (2006). Towards understanding of glycaemic index and glycaemic load in habitual diet: Associations with measures of glycaemia in the insulin resistance atherosclerosis study. Br. J. Nutr..

[66] Hodge A.M., English D.R., O’Dea K., Giles G.G. (2004). Glycemic index and dietary fiber and the risk of type 2 diabetes. Diabetes Care.

[67] Salmerón J., Ascherio A., Rimm E.B., Colditz G.A., Spiegelman D., Jenkins D.J.A., Stampfer M.J., Wing A.L., Willett W.C. (1997). Dietary fiber, glycemic load, and risk of NIDDM in men. Diabetes Care.

[68] Runchey S.S., Pollak M.N., Valsta L.M., Coronado G.D., Schwarz Y., Breymeyer K.L., Wang C., Wang C.Y., Lampe J.W., Neuhouser M.L. (2012). Glycemic load effect on fasting and post-prandial serum glucose, insulin, IGF-1 and IGFBP-3 in a randomized, controlled feeding study. Eur. J. Clin. Nutr..

[69] Ojo O., Ojo O.O., Adebowale F., Wang X.H. (2018). The effect of dietary glycaemic index on glycaemia in patients with type 2 diabetes: A systematic review and meta-analysis of randomized controlled trials. Nutrients.

[70] Bhupathiraju S.N., Tobias D.K., Malik V.S., Pan A., Hruby A., Manson J.E., Willett W.C., Hu F.B. (2014). Glycemic index, glycemic load, and risk of type 2 diabetes: Results from 3 large US cohorts and an updated meta-analysis. Am. J. Clin. Nutr..

[71] Dong J.Y., Zhang L., Zhang Y.H., Qin L.Q. (2011). Dietary glycaemic index and glycaemic load in relation to the risk of type 2 diabetes: A meta-analysis of prospective cohort studies. Br. J. Nutr..

[72] Livesey G., Taylor R., Livesey H., Liu S. (2013). Is there a dose-response relation of dietary glycemic load to risk of type 2 diabetes? Meta-analysis of prospective cohort studies. Am. J. Clin. Nutr..

[73] Yu D., Shu X.O., Li H., Xiang Y.B., Yang G., Gao Y.T., Zheng W., Zhang X. (2013). Dietary carbohydrates, refined grains, glycemic load, and risk of coronary heart disease in Chinese adults. Am. J. Epidemiol..

[74] Burger K.N., Beulens J.W., Boer J.M., Spijkerman A.M., Van Der A.D. (2011). Dietary glycemic load and glycemic index and risk of coronary heart disease and stroke in Dutch men and women: The EPIC-MORGEN study. PLoS ONE.

[75] Sieri S., Krogh V., Berrino F., Evangelista A., Agnoli C., Brighenti F., Pellegrini N., Palli D., Masala G., Sacerdote C. (2010). Dietary glycemic load and index and risk of coronary heart disease in a large Italian cohort: The EPICOR study. Arch. Intern. Med..

[76] Levitan E.B., Mittleman M.A., Hakansson N., Wolk A. (2007). Dietary glycemic index, dietary glycemic load, and cardiovascular disease in middle-aged and older Swedish men. Am. J. Clin. Nutr..

[77] Burger K.N., Beulens J.W., Van Der Schouw Y.T., Sluijs I., Spijkerman A.M., Sluik D., Boeing H., Kaaks R., Teucher B., Dethlefsen C. (2012). Dietary fiber, carbohydrate quality and quantity, and mortality risk of individuals with diabetes mellitus. PLoS ONE.

[78] Levitan E.B., Mittleman M.A., Wolk A. (2010). Dietary glycemic index, dietary glycemic load, and incidence of heart failure events: A prospective study of middle-aged and elderly women. J. Am. Coll. Nutr..

[79] Levitan E.B., Cook N.R., Stampfer M.J., Ridker P.M., Rexrode K.M., Buring J.E., Manson J.E., Liu S. (2008). Dietary glycemic index, dietary glycemic load, blood lipids, and c-reactive protein. Metabolism.

[80] Liese A.D., Gilliard T., Schulz M., D’Agostino R.B., Wolever T.M. (2007). Carbohydrate nutrition, glycaemic load, and plasma lipids: The insulin resistance atherosclerosis study. Eur. Heart J..

[81] Milton J.E., Briche B., Brown I.J., Hickson M., Robertson C.E., Frost G.S. (2007). Relationship of glycaemic index with cardiovascular risk factors: Analysis of the national diet and nutrition survey for people aged 65 and older. Public Health Nutr..

[82] Castro-Quezada I., Artacho R., Molina-Montes E., Serrano F.A., Ruiz-Lopez M.D. (2015). Dietary glycaemic index and glycaemic load in a rural elderly population (60–74 years of age) and their relationship with cardiovascular risk factors. Eur. J. Nutr..

[83] Murakami K., Sasaki S., Takahashi Y., Okubo H., Hosoi Y., Horiguchi H., Oguma E., Kayama F. (2006). Dietary glycemic index and load in relation to metabolic risk factors in Japanese female farmers with traditional dietary habits. Am. J. Clin. Nutr..

[84] Shikany J.M., Tinker L.F., Neuhouser M.L., Ma Y., Patterson R.E., Phillips L.S., Liu S., Redden D.T. (2010). Association of glycemic load with cardiovascular disease risk factors: The women’s health initiative observational study. Nutrition.

[85] Hosseinpour-Niazi S., Sohrab G., Asghari G., Mirmiran P., Moslehi N., Azizi F. (2013). Dietary glycemic index, glycemic load, and cardiovascular disease risk factors: Tehran lipid and glucose study. Arch. Iran. Med..

[86] McKeown N.M., Meigs J.B., Liu S., Rogers G., Yoshida M., Saltzman E., Jacques P.F. (2009). Dietary carbohydrates and cardiovascular disease risk factors in the framingham offspring cohort. J. Am. Coll. Nutr..

[87] Masson C.J., Mensink R.P. (2011). Exchanging saturated fatty acids for (n-6) polyunsaturated fatty acids in a mixed meal may decrease postprandial lipemia and markers of inflammation and endothelial activity in overweight men. J. Nutr..

[88] Mekki N., Charbonnier M., Borel P., Leonardi J., Juhel C., Portugal H., Lairon D. (2002). Butter differs from olive oil and sunflower oil in its effects on postprandial Lipemia and Triacylglycerol-rich lipoproteins after single mixed meals in healthy young men. J. Nutr..

[89] Peairs A.D., Rankin J.W., Lee Y.W. (2011). Effects of acute ingestion of different fats on oxidative stress and inflammation in overweight and obese adults. Nutr. J..

[90] Tulk H.M.F., Robinson L.E. (2009). Modifying the n-6/n-3 polyunsaturated fatty acid ratio of a high–saturated fat challenge does not acutely attenuate postprandial changes in inflammatory markers in men with metabolic syndrome. Metab. Clin. Exp..

[91] Westphal S., Leodolter A., Kahl S., Dierkes J., Malfertheiner P., Luley C. (2002). Addition of glucose to a fatty meal delays chylomicron and suppresses VLDL in healthy subjects. Eur. J. Clin. Investig..

[92] Westphal S., Kastner S., Taneva E., Leodolter A., Dierkes J., Luley C. (2004). Postprandial lipid and carbohydrate responses after the ingestion of a casein-enriched mixed meal. Am. J. Clin. Nutr..

[93] Brannon P.M. (1990). Adaptation of the exocrine pancreas to diet. Annu. Rev. Nutr..

[94] Armand M., Hamosh M., DiPalma J.S., Gallagher J., Benjamin S.B., Philpott J.R., Lairon D., Hamosh P. (1995). Dietary fat modulates gastric lipase activity in healthy humans. Am. J. Clin. Nutr..

[95] Pasquier B., Armand M., Guillon F., Castelain C., Borel P., Barry J.L., Pieroni G., Lairon D. (1996). Viscous soluble dietary fibers alter emulsification and lipolysis of triacylglycerols in duodenal medium in vitro. J. Nutr. Biochem..

[96] Ausar S.F., Landa C.A., Bianco I.D., Castagna L.F., Beltramo D.M. (2001). Hydrolysis of a chitosan-induced milk aggregate by pepsin, trypsin and pancreatic lipase. Biosci. Biotechnol. Biochem..

[97] D’Souza V.M., Shertzer H.G., Menon A.G., Pauletti G.M. (2003). High glucose concentration in isotonic media alters caco-2 cell permeability. AAPS PharmSci.

[98] D’Souza V.M., Buckley D.J., Buckley A.R., Pauletti G.M. (2003). Extracellular glucose concentration alters functional activity of the intestinal oligopeptide transporter (PepT-1) in Caco-2 cells. J. Pharm. Sci..

[99] Yamamoto M., Acevedo-Duncan M., Chalfant C.E., Patel N.A., Watson J.E., Cooper D.R. (2000). Acute glucose-induced downregulation of PKCbetaII accelerates cultured VSMC proliferation. Am. J. Physiol. Cell Physiol..

[100] Abe-Dohmae S., Ikeda Y., Matsuo M., Hayashi M., Okuhira K., Ueda K., Yokoyama S. (2004). Human ABCA7 supports apolipoprotein-mediated release of cellular cholesterol and phospholipid to generate high density lipoprotein. J. Biol. Chem..

[101] Kobayashi T., Ogawa Y., Watanabe Y., Furuya M., Kataoka S., Garcia del Saz E., Tsunawaki S., Dinauer M.C., Seguchi H. (2004). Mitochondrial transmembrane potential is diminished in phorbol myristate acetate-stimulated peritoneal resident macrophages isolated from wild-type mice, but not in those from gp91-phoxdeficient mice. Histochem. Cell Biol..

[102] Iizuka K., Bruick R.K., Liang G., Horton J.D., Uyeda K. (2004). Deficiency of carbohydrate response element-binding protein (ChREBP) reduces lipogenesis as well as glycolysis. Proc. Natl. Acad. Sci. USA.

[103] Aragno M., Tomasinelli C.E., Vercellinatto I., Catalano M.G., Collino M., Fantozzi R., Danni O., Boccuzzi G. (2009). SREBP-1c in nonalcoholic fatty liver disease induced by western-type high-fat diet plus fructose in rats. Free Radic. Biol. Med..

[104] Bidwell A.J. (2017). Chronic Fructose Ingestion as a Major Health Concern: Is a Sedentary Lifestyle Making It Worse? A Review. Nutrients.

[105] Tappy L., Randin J.P., Felber J.P., Chiolero R., Simonson D.C., Jequier E., DeFronzo R.A. (1986). Comparison of thermogenic effect of fructose and glucose in normal humans. Am. J. Physiol..

[106] Stanhope K.L., Havel P.J. (2008). Fructose consumption: Potential mechanisms for its effects to increase visceral adiposity and induce dyslipidemia and insulin resistance. Curr. Opin. Lipidol..

[107] Sun S.Z., Empie M.W. (2012). Fructose metabolism in humans—What isotopic tracer studies tell us. Nutr. Metab..

[108] Chalasani N., Younossi Z., Lavine J.E., Diehl A.M., Brunt E.M., Cusi K., Charlton M., Sanyal A.J. (2012). The diagnosis and management of non-alcoholic fatty liver disease: Practice guideline by the American Gastroenterological Association, American Association for the Study of Liver Diseases, and American College of Gastroenterology. Gastroenterology.

[109] Mayes P.A. (1993). Intermediary metabolism of fructose. Am. J. Clin. Nutr..

[110] Ma J., Fox C., Speliotes E., Hoffmann U., Smith C., Saltzman E., Jacques P., McKeown N. (2015). Sugar-sweetened beverage intake is associated with fatty liver in the Framingham offspring study. J. Hepatol..

[111] Ouyang X., Cirillo P., Sautin Y., McCall S., Bruchette J.L., Diehl A.M., Johnson R.J., Abdelmalek M.F. (2008). Fructose consumption as a risk factor for non-alcoholic fatty liver disease. J. Hepatol..

[112] Kanerva N., Sandboge S., Kaartinen N.E., Mannisto S., Eriksson J.G. (2014). Higher fructose intake is inversely associated with risk of nonalcoholic fatty liver disease in older Finnish adults. Am. J. Clin. Nutr..

[113] Abdelmalek M.F., Suzuki A., Guy C., Unalp-Arida A., Colvin R., Johnson R.J., Diehl A.M. (2010). Increased fructose consumption is associated with fibrosis severity in patients with NAFLD. Hepatology.

[114] Chung M., Ma J., Patel K., Berger S., Lau J., Lichtenstein A.H. (2014). Fructose, high-fructose corn syrup, sucrose, and nonalcoholic fatty liver disease or indexes of liver health: A systematic review and meta-analysis. Am. J. Clin. Nutr..

[115] Chiu S., Sievenpiper J.L., De Souza R.J., Cozma A.I., Mirrahimi A., Carleton A.J., Ha V., Di Buono M., Jenkins A.L., Leiter L.A. (2014). Effect of fructose on markers of non-alcoholic fatty liver disease (NAFLD): A systematic review and meta-analysis of controlled feeding trials. Eur. J. Clin. Nutr..

[116] Ter Horst K.W., Serlie M.J. (2017). Fructose Consumption, Lipogenesis, and Non-Alcoholic Fatty Liver Disease. Nutrients.

[117] Matikainen N., Söderlund S., Björnson E., Bogl L.H., Pietiläinen K.H., Hakkarainen A., Lundbom N., Eliasson B., Räsänen S.M., Rivellese A. (2017). Fructose intervention for 12 weeks does not impair glycemic control or incretin hormone responses during oral glucose or mixed meal tests in obese men. Nutr. Metab. Cardiovasc. Dis..

[118] Swarbrick M.M., Stanhope K.L., Elliott S.S., Graham J.L., Krauss R.M., Christiansen M.P., Griffen S.C., Keim N.L., Havel P.J. (2008). Consumption of fructose-sweetened beverages for 10 weeks increases postprandial triacylglycerol and apolipoprotein-B concentrations in overweight and obese women. Br. J. Nutr..

[119] Stanhope K.L., Griffen S.C., Bremer A.A., Vink R.G., Schaefer E.J., Nakajima K., Schwarz J.-M., Beysen C., Berglund L., Keim N.L. (2011). Metabolic responses to prolonged consumption of glucose- and fructose-sweetened beverages are not associated with postprandial or 24-h glucose and insulin excursions. Am. J. Clin. Nutr..

[120] Chong M.F., Fielding B.A., Frayn K.N. (2007). Mechanisms for the acute effect of fructose on postprandial lipemia. Am. J. Clin. Nutr..

[121] Bantle J.P., Raatz S.K., Thomas W., Georgopoulos A. (2000). Effects of dietary fructose on plasma lipids in healthy subjects. Am. J. Clin. Nutr..

[122] Stanhope K.L., Schwarz J.M., Keim N.L., Griffen S.C., Bremer A.A., Graham J.L., Hatcher B., Cox C.L., Dyachenko A., Zhang W. (2009). Consuming fructose-sweetened, not glucose-sweetened, beverages increases visceral adiposity and lipids and decreases insulin sensitivity in overweight/obese humans. J. Clin. Investig..

[123] Harbis A., Perdreau S., Vincent-Baudry S., Charbonnier M., Bernard M.C., Raccah D., Senft M., Lorec A.M., Defoort C., Portugal H. (2004). Glycemic and insulinemic meal responses modulate postprandial hepatic and intestinal lipoprotein accumulation in obese, insulin-resistant subjects. Am. J. Clin. Nutr..

[124] Harbis A., Defoort C., Narbonne H., Juhel C., Senft M., Latgé C., Delenne B., Portugal H., Atlan-Gepner C., Vialettes B. (2001). Acute hyperinsulinism modulates plasma apolipoprotein B-48 triglyceride-rich lipoproteins in healthy subjects during the postprandial period. Diabetes.

[125] Bouché C., Rizkalla S.W., Luo J., Vidal H., Veronese A., Pacher N., Fouquet C., Lang V., Slama G. (2002). Five-week, low-glycemic index diet decreases total fat mass and improves plasma lipid profile in moderately overweight nondiabetic men. Diabetes Care.

[126] Bukkapatnam R.N., Berglund L., Anuurad E., Devaraj S., Hyson D., Rafii F., Malmstein C., Villablanca A.C. (2010). Postprandial metabolic responses to dietary glycemic index in hypercholesterolemic postmenopausal women. Prev. Cardiol..

[127] Sun L., Tan K.W.J., Lim J.Z., Magkos F., Henry C.J. (2018). Dietary fat and carbohydrate quality have independent effects on postprandial glucose and lipid responses. Eur. J. Nutr..

[128] Despland C., Walther B., Kast C., Campos V., Rey V., Stefanoni N., Tappy L. (2017). A randomized-controlled clinical trial of high fructose diets from either Robinia honey or free fructose and glucose in healthy normal weight males. Clin. Nutr. ESPEN.

[129] Campos V., Despland C., Brandejsky V., Kreis R., Schneiter P., Boesch C., Tappy L. (2017). Metabolic Effects of Replacing Sugar-Sweetened Beverages with Artificially-Sweetened Beverages in Overweight Subjects with or without Hepatic Steatosis: A Randomized Control Clinical Trial. Nutrients.

[130] Livesey G., Taylor R. (2008). Fructose consumption and consequences for glycation, plasma triacylglycerol, and body weight: Meta-analyses and meta-regression models of intervention studies. Am. J. Clin. Nutr..

[131] David Wang D., Sievenpiper J.L., De Souza R.J., Cozma A.I., Chiavaroli L., Ha V., Mirrahimi A., Carleton A.J., Di Buono M., Jenkins A.L. (2014). Effect of fructose on postprandial triglycerides: A systematic review and meta-analysis of controlled feeding trials. Atherosclerosis.

